# Inhibition of Inflammatory Cytokine Expression Prevents High-Fat Diet-Induced Kidney Injury: Role of Lingonberry Supplementation

**DOI:** 10.3389/fmed.2020.00080

**Published:** 2020-03-27

**Authors:** Susara Madduma Hewage, Suvira Prashar, Samir C. Debnath, Karmin O, Yaw L. Siow

**Affiliations:** ^1^Canadian Centre for Agri-Food Research in Health and Medicine, St. Boniface Hospital Albrechtsen Research Centre, Winnipeg, MB, Canada; ^2^Department of Physiology & Pathophysiology, University of Manitoba, Winnipeg, MB, Canada; ^3^Agriculture and Agri-Food Canada, St. Boniface Hospital Albrechtsen Research Centre, Winnipeg, MB, Canada; ^4^Agriculture and Agri-Food Canada, St. John's Research and Development Centre, St. John's, NL, Canada; ^5^Department of Animal Science, University of Manitoba, Winnipeg, MB, Canada

**Keywords:** chronic kidney disease, high-fat diet, lingonberry, cytokines, inflammation, NF-κB

## Abstract

Chronic low-grade inflammation is a major stimulus for progression of chronic kidney disease (CKD) in individuals consuming high-fat diet. Currently, there are limited treatment options for CKD other than controlling the progression rate and its associated complications. Lingonberry (*Vaccinium vitis-idaea* L.) is rich in anthocyanins with demonstrated anti-inflammatory effect. In the current study, we investigated the potential renal protective effect of lingonberry and its anthocyanin (cyanidin-3-glucoside) in high-fat diet fed obese mice and in human proximal tubular cells. Prolonged consumption of high-fat diets is strongly associated with obesity, abnormal lipid and glucose metabolism. Mice (C57BL/6J) fed a high-fat diet (62% kcal fat) for 12 weeks developed renal injury as indicated by an elevation of blood urea nitrogen (BUN) level as well as an increase in renal kidney injury molecule-1 (KIM-1), neutrophil gelatinase-associated lipocalin (NGAL) and renin expression. Those mice displayed an activation of nuclear factor kappa-light-chain-enhancer of activated B cells (NF-κB) and increased expression of inflammatory cytokines–monocyte chemoattractant-1 (MCP-1), tumor necrosis factor alpha (TNF-α), interleukin-6 (IL-6) in the kidneys. Mice fed a high-fat diet also had a significant elevation of inflammatory cytokine levels in the plasma. Dietary supplementation of lingonberry for 12 weeks not only attenuated high-fat diet-induced renal inflammatory response but also reduced kidney injury. Such a treatment improved plasma lipid and glucose profiles, reduced plasma inflammatory cytokine levels but did not affect body weight gain induced by high-fat diet feeding. Lingonberry extract or its active component cyanidin-3-glucoside effectively inhibited palmitic acid-induced NF-κB activation and inflammatory cytokine expression in proximal tubular cells. These results suggest that lingonberry supplementation can reduce inflammatory response and prevent chronic kidney injury. Such a renal protective effect by lingonberry and its active component may be mediated, in part, through NF-κB signaling pathway.

## Introduction

Chronic kidney disease (CKD) is a common kidney disease with a progressive decline of renal function (1). Obesity and metabolic syndrome are independent risk factors for the development of CKD (2). Obesity is prevalent in many countries around the world. Large cohort studies have shown that the incidence of CKD increases by 20–88% in obese individuals (3–5). There is increasing evidence that patients who survived acute kidney injury have increased risks in developing CKD (6). CKD has emerged as a serious economic threat to health care systems globally due to its increasing prevalence, complications (such as anemia, cardiovascular disease, bone, and mineral disorders), immense expenses associated with renal replacement therapy, high morbidity and mortality (1).

The pathophysiology of CKD is complex and incompletely understood. Several mechanisms by which obesity causes CKD have been proposed, which include renal lipid accumulation, inflammation and mitochondrial dysfunction (5, 7, 8). Chronic inflammatory response has been implicated as one of the important mediators contributing to kidney injury in patients with obesity (9). A study conducted in patients with chronic renal insufficiency revealed that kidney injury was positively correlated to the levels of proinflammatory cytokines, namely, tumor necrosis factor alpha (TNF-α) and interleukin-6 (IL-6) in the plasma (10). It was reported that casein-induced inflammatory stress promoted renal lipid accumulation and glomerular lesion formation in high-fat diet fed obese mice that displayed renal and systemic changes compatible to human obesity-related CKD (8). Chronic consumption of high-fat diets (HFD) is a major contributor to the development of obesity and metabolic abnormalities. In our previous studies, we observed an increased body weight gain and metabolic abnormalities (hyperlipidemia, hyperglycemia) in mice fed a HFD for 5–12 weeks (11–16). Recent studies demonstrated renal injury in diet-induced obese mice, a murine model of CKD (7, 17).

Currently, there is no specific treatment for CKD other than lowering the progression rate by controlling the CKD risk factors and its associated complications (edema, anemia, cardiovascular diseases, and bone and mineral disorders) (18). The most common medications prescribed for CKD patients include diuretics, erythropoietins, antihypertensive agents, statins and calcium and vitamin D supplements (18). Certain dietary restrictions are recommended to control the intake of protein, sugar, salt, and fiber content (18). In our previous study, we reported that supplementation of diet with lingonberry juice could protect rats against ischemia-reperfusion-induced acute kidney injury (19). Lingonberry (*Vaccinium vitis-idaea* L.) is an evergreen dwarf shrub native to North America and Eurasia throughout the Northern Hemisphere (20). The bright reddish color berries produced by these plants are edible and is opulent with anthocyanins compared to other commonly consumed berries (21). Anthocyanins are a group of water soluble flavonoids known for their antioxidant, anti-inflammatory, and anticancer properties (22). Lingonberry contains three types of anthocyanins, identified as cyanidin-3-galactoside, cyanidin-3-glucoside (C-3-Glu) and cyanidin-3-arabinoside (23). Among these, C-3-Glu is known to improve the redox status, energy, and glucose metabolism in rat kidneys (24). Furthermore, recent studies have revealed that lingonberry exhibits antidiabetic and renal protective properties (19, 25). However, its role in CKD has not been identified. In the current study, we investigated the potential renal protective effect of lingonberry and its anthocyanin (C-3-Glu) in HFD-induced obese mice and in human proximal tubular cells.

## Materials and Methods

### Animal Model

Five weeks old C57BL/6J male mice were purchased from the University of Manitoba Central Animal Care Services (Winnipeg, MB, Canada). Animals were housed two per cage in a temperature- and humidity-controlled room with a 12 h dark−12 h light cycle, and freely accessible for water and feed. Mice were divided into three groups (*n* = 10): (1) Control (D12450J) diet consisted of 11% kcal fat, 18% kcal protein, and 71% kcal carbohydrate, (2) HFD (D12492) consisted of 62% kcal fat, 18% kcal protein, and 20% kcal carbohydrate, and (3) HFD supplemented with 5% Manitoba lingonberry (*Vaccinium vitis-idaea* ssp. *minus* (Lodd.) Hult.) (w/w) (D17022206). The animals were fed *ad libitum* for 12 weeks with the diets listed (Research Diets, Brunswick, NJ, USA). Fasting blood was collected at week 12 from the jugular vein after a 6 h fasting period to measure the following plasma analytes: triglyceride, total cholesterol, and urea/BUN, using the Cobas C111 Analyzer (Roche, Risch-Rotkreuz, Switzerland). Fasting blood glucose was measured at week 12 using AlphaTRAK 2 Blood Glucose Test Strips (Zoetis, Kirkland, QC, Canada). At the end of week 12, animals were sacrificed and their kidneys and blood were collected. All procedures were performed in accordance with the Guide to the Care and Use of Experimental Animals published by the Canadian Council on Animal Care and approved by the University of Manitoba Protocol Management and Review Committee.

### Cell Culture

Human proximal tubule epithelial cells (HK-2) were purchased from American Type Culture Collection (CRL-2190, Manassas, VA, USA) and maintained in keratinocyte serum-free media supplemented with 5 ng/ml human recombinant epidermal growth factor and 50 ng/ml bovine pituitary extract (Life Technologies, Carlsbad, CA, USA) at 37°C in 95% oxygen−5% carbon dioxide atmosphere. The cells were sub-cultured at or below 90% confluency once every 2–3 days and passages between 5 and 20 were used for all the experiments. Depending on the assay, cells were seeded in 6-well plates or 100 mm dishes at a density of 2.0 × 10^5^ cells per well or 2 × 10^6^ cells per dish. Cells seeded in 6-well plate were pre-treated for 4 h either with cyanidin-3-glucoside (C-3-Glu) (2, 10, and 20 μM; Cerilliant Corp., Round Rock, TX, USA) or various dilutions of lingonberry extract (1:1,000, 1:500, and 1:200). The lingonberry (LB) extract was prepared as previously described (23). After the pre-treatment, the cells were incubated for 4 h with palmitic acid (Sigma-Aldrich. St. Louis, MO, USA; dissolved in 10% fatty acid-free bovine serum albumin by gently shaking overnight at 37°C) prior to being added to the culture medium (16). In one set of experiments, cells were pre-incubated for 1 h with 100 μM ammonium pyrrolidinedithiocarbamate (PDTC; Sigma Aldrich), a selective inhibitor of NF-κB, prior to treatment with palmitic acid.

### Cell Viability Assay

The influence of palmitic acid, C-3-Glu and LB extract on HK-2 cell viability was examined using the 3-(4,5-dimethylthiazol-2-yl)-2,5-diphenyltetrazolium bromide (MTT) assay. Cells were seeded in a 96-well plate at a density of 20,000 cells/well. After 24 h incubation, cells were treated with different concentrations of palmitic acid, C-3-Glu or LB extract for another 24 h. The yellow tetrazolium MTT (Sigma-Aldrich) was added to each well to yield a final concentration of 100 μM. The supernatant was aspirated 4 h later and the MTT formazan crystals were dissolved in dimethyl sulfoxide (DMSO; Thermo Fisher Scientific, Waltham, MA, USA). The absorbance at 540 nm was read using a SpectraMax M5 microplate reader (Molecular Devices, Sunnyvale, CA, USA).

### Measurement of mRNA Expression

Relative mRNA expression of kidney injury molecule-1 (KIM-1), IL-6, monocyte chemoattractant protein-1 (MCP-1), neutrophil gelatinase-associated lipocalin (NGAL), renin and TNF-α, were measured using a StepOne Plus Real-Time qPCR (RT-qPCR) system (Applied Biosystems, Foster City, CA, USA). Briefly, total RNA was extracted from the HK-2 cells and the mouse kidney tissues with TRIzol (Thermo Fisher Scientific) and QIAzol (Qiagen, Hilden, Germany) reagents, respectively. HK-2 cells grown in 6-well plates were washed twice with ice cold PBS and the cell lysate was collected by adding 1 ml of TRIzol per well. Kidney tissues preserved in RNA*later* (Thermo Fisher Scientific) were homogenized with a handheld homogenizer (VWR 200, VWR, Radnor, PA, USA) in 1 ml of QIAzol reagent on ice. Total RNA was extracted according to the TRIzol reagent procedure for isolation of RNA as described by Chomczynski and Mackey (26). Extracted RNA was quantified using a NanoDrop One^C^ (Thermo Fisher Scientific) spectrophotometer. cDNA was synthesized by mixing 1X first-strand buffer, 10 mM dithiothreitol (DTT), 0.5 mM deoxynucleotide triphosphates (dNTPs), 10 ng/μl Oligo dT primers, 1 U/μl RNaseOUT recombinant ribonuclease inhibitor, 1 U/μl moloney murine leukemia virus (M-MLV) reverse transcriptase (Thermo Fisher Scientific), with 1 μg of total RNA in a total volume of 20 μl. Then the mixture was incubated for 60 min at 37°C and 2 min at 95°C. The RT-qPCR mixture contained 100 ng of cDNA, 1X iTaq Universal SYBR Green Super Mix (Bio-Rad, Hercules, CA, USA), 300 nM per primer and RNase-free water, in a total reaction mixture of 20 μl. The qPCR protocol was initiated as follows: the reaction mixture was subjected to initial denaturation at 95°C for 3 min followed by 45 cycles of denaturation step 95°C, 10 s and annealing. Annealing temperatures for TNF-α, IL-6, MCP-1, NGAL and renin were set at 58°C for 30 s while for KIM-1, it was set at 60°C for 15 s. For each primer a melt curve analysis was performed. All the samples were tested in triplicate and data were analyzed using the comparative C_T_ method (27) with gene expression level normalized to that of the housekeeping gene β-actin. The results were expressed as a percentage of the control group, which was set to 100%. Primer sequences used for the RT-qPCR were shown in the Table 1.

**Table 1:** Primer sequences used for RT-qPCR.

**Primer**	**Sequence 5′-3′**	**Accession number**	**Product length**
**Human**			
IL-6	F: ACTCACCTCTTCAGAACGAATTG	XM_005249745.5	149 bp
	R: CCATCTTTGGAAGGTTCAGGTTG		
MCP-1	F: CCCAAAGAAGCTGTGATCTTCA	NM_002982.4	186 bp
	R: GTGTCTGGGGAAAGCTAGGG		
TNF-α	F: GAGGCCAAGCCCTGGTATG	NM_000594.4	91 bp
	R: CGGGCCGATTGATCTCAGC		
β-Actin	F: AGATCAAGATCATTGCTCCTCCT	NM_001101.5	95 bp
	R: GATCCACATCTGCTGGAAGG		
**Mouse**			
IL-6	F: GACTGATGCTGGTGACAACC	NM_001314054.1	170 bp
	R: GCCATTGCACAACTCTTTTC		
MCP-1	F: AGGTCCCTGTCATGCTTCTG	NM_011333.3	167 bp
	R: GCTGCTGGTGATCCTCTTGT		
TNF-α	F: GTCCCCAAAGGGATGAGAAG	NM_001278601.1	93 bp
	R: GCTCCTCCACTTGGTGGTTT		
NGAL	F: ACGGACTACAACCAGTTCGC	NM_008491.1	192 bp
	R: AATGCATTGGTCGGTGGGG		
KIM-1	F: TCCACACATGTACCAACATCAA	XM_011248784.2	98 bp
	R: GTCACAGTGCCATTCCAGTC		
β-Actin	F: GATCAAGATCATTGCTCCTCCT	XM_030254057.1	183 bp
	R: AGGGTGTAAAACGCAGCTCA		

### Electrophoretic Mobility Shift Assay (EMSA)

LightShift Chemiluminescent EMSA Kit (Thermo Fisher Scientific) was used to measure the DNA binding affinity of NF-κB. In brief, nuclear proteins were extracted from the mouse kidney tissues and HK-2 cells as previously described (28). Nuclear proteins (2 μg) were incubated in a reaction mixture containing DNA-binding buffer, poly (dI-dC), and biotin-end-labeled oligonucleotides containing a consensus sequence specific for the NF-κB-binding site (5′-AGTTGAGGGGACTTTCCAGGC-3′) (Promega, Madison, WI, USA), according to manufacturer's instructions. The NF-κB oligonucleotide was labeled with biotin at the 3′ end using the Pierce Biotin 3′ End DNA labeling kit (Thermo Fisher Scientific). Following incubation, reaction mixtures were loaded in a 6% non-denaturing polyacrylamide gel to facilitate separation of DNA-protein complexes and transferred to a nylon membrane (Thermo Fisher Scientific) for detection using the Chemiluminescent Nucleic Acid Detection Module kit (Thermo Fisher Scientific).

### Immunoblotting

An aliquot of the nuclear proteins (10 μg) prepared for EMSA was subjected to Western immunoblotting analysis (28, 29). Briefly, the nuclear proteins were separated by electrophoresis in a 12% SDS-polyacrylamide gel and transferred onto nitrocellulose membrane (Bio-Rad) using a Trans-Blot Turbo Transfer System (Bio-Rad). The membranes were probed with anti-histone H3 antibody (SC-10809; Santa Cruz Biotechnology Inc., Dallas, TX, USA).

### Proinflammatory Marker Analysis

Plasma TNF-α, IL-6, and MCP-1 protein levels were measured using a U-PLEX Biomarker Group 1 kit (MesoScale Discovery, Rockville, MD, USA). Briefly, an aliquot of plasma (25 μl) was loaded into a plate containing pre-coated biotinylated antibodies for specific inflammatory marker. The assay was performed according to the manufacturer's instructions and quantitative chemiluminescence data was obtained using the QuickPlex SQ 120 (MesoScale Discovery) followed by analysis using the Discovery Workbench 4.0 Software (MesoScale Discovery).

### Histological Analysis and Immunohistochemistry

A portion of the kidney was immersion-fixed overnight in 10% neutral buffered formalin (10% formalin, 25 mM NaH_2_PO_4_, 45 mM Na_2_HPO_4_) and then embedded in paraffin (29). Paraffin-embedded sections were cut at a thickness of 5 μm and stained with hematoxylin and eosin (H&E) to evaluate the morphological changes. Paraffin embedded sections were used for immunostaining. In brief, tissue sections were boiled in EDTA antigen repairing buffer. The sections were naturally cooled and incubated with 3% hydrogen peroxide solution at room temperature for 15 min to block the endogenous peroxidase activity. Slides were incubated overnight at 4°C with anti-F4/80 antibody (1:100 dilution, MCA497, Bio-Rad) or anti-MPO antibody (1:100 dilution, ab9535, Abcam, Cambridge, United Kingdom) in a humidified chamber. The sections were then incubated for 1 h with biotinylated goat anti-rat IgG or goat anti-rabbit IgG (1:200, Dako, Glostrup, Denmark), respectively, followed by incubation with streptavidin-horse radish peroxidase (HRP) conjugate (Zymed Laboratories, Inc., San Francisco, CA, USA). Finally, the slides were counterstained with Mayer's hematoxylin. For the negative controls, normal rabbit IgG and rat IgG were used as primary antibodies. All images were captured using an Olympus BX43 Upright Light Microscope (Olympus Corp., Tokyo, Japan) equipped with a Q-color 3 digital camera and analyzed using Image-Pro plus 7.0 (Media Cybernetics, Rockville, MD, USA).

### Statistical Analysis

Data are presented as means ± standard error. Results were analyzed using one-way ANOVA followed by Newman–Keuls multiple comparison test. *P* < 0.05 were considered statistically significant. All statistical analyses were performed ProStat Version 6 software (Poly Software International, Pearl River, NY, USA).

## Results

### Effect of HFD and Lingonberry Supplementation on Body Weight, Kidney Function and Metabolic Parameters

**Figure 1:**
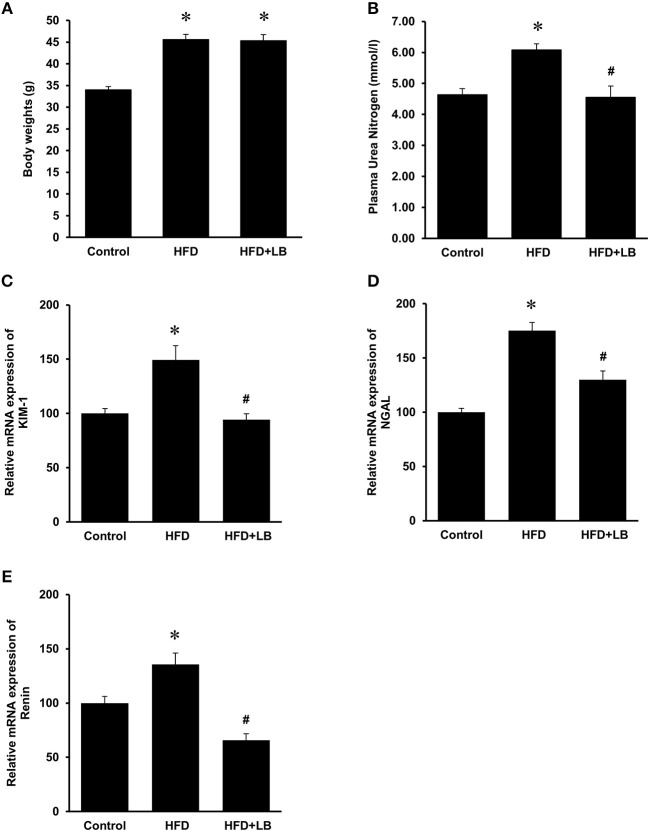
Body weights and kidney injury parameters. Mice were fed a control diet, high-fat diet (HFD) or HFD supplemented with lingonberry (HFD+LB) for 12 weeks and **(A)** final body weights were measured. Kidney injury was examined by measuring **(B)** blood urea nitrogen (BUN), renal mRNA expression of **(C)** kidney injury molecule-1 (KIM-1), **(D)** neutrophil gelatinase-associated lipocalin (NGAL) and **(E)** renin. The results are expressed as the means ± SE (*n* = 6). *p < 0.05 when compared with the value obtained from the control group. ^#^*p* < 0.05 when compared with the value obtained from the HFD group.

**Figure 2:**
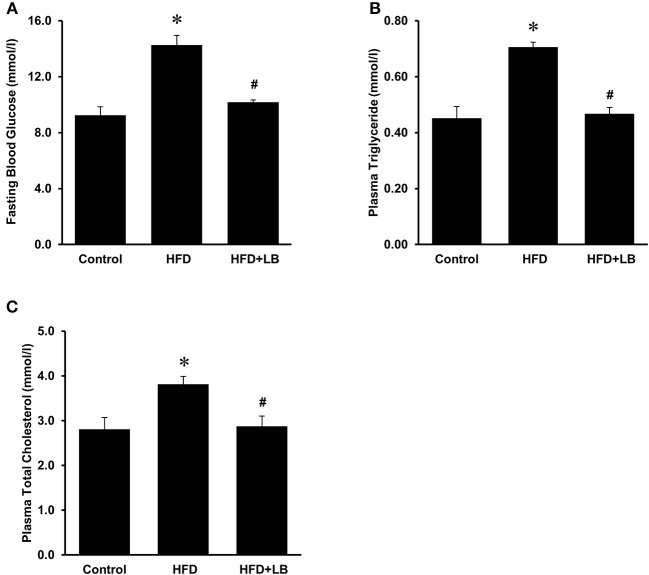
Blood glucose and lipid profile in mice. Mice were fed a control diet, high-fat diet (HFD) or HFD supplemented with lingonberry (HFD+LB) for 12 weeks. **(A)** Blood glucose was measured after fasting for 6 h. Plasma **(B)** triglyceride and **(C)** total cholesterol were also measured. The results are expressed as the means ± SE (*n* = 6). *p < 0.05 when compared with the value obtained from the control group. ^#^*p* < 0.05 when compared with the value obtained from the HFD group.

The initial average body weights of the mice ranged from 22.5–23.0 g. Feeding mice a HFD for 12 weeks caused a significant elevation in body weight as compared to mice fed a control diet (Figure 1A). Supplementation of lingonberry for 12 weeks did not change the body weight gain induced by the HFD feeding (Figure 1A). Kidney function was assessed by measuring BUN levels in the plasma, gene expression of KIM-1, NGAL and renin in the kidneys. HFD feeding resulted in a significant elevation of BUN levels in the plasma (Figure 1B) and a significant increase in the expression of KIM-1, NGAL, and renin mRNA in the kidneys (Figures 1C–E), indicating kidney function was impaired. Lingonberry supplementation reduced plasma BUN levels as well as renal KIM-1, NGAL and renin gene expression (Figure 1). Mice fed a HFD had a higher level of fasting blood glucose than the control group (Figure 2A). Lingonberry supplementation significantly reduced the fasting blood glucose level in mice fed a HFD (Figure 2A). There was also a significant increase in plasma levels of triglycerides (Figure 2B) and total cholesterol (Figure 2C) in mice fed a HFD. Lingonberry supplementation reduced plasma triglyceride and cholesterol levels to that similar to the control group (Figures 2B,C).

### Effect of HFD and Lingonberry Supplementation on Inflammatory Cytokine Expression in the Kidneys and Plasma

**Figure 3:**
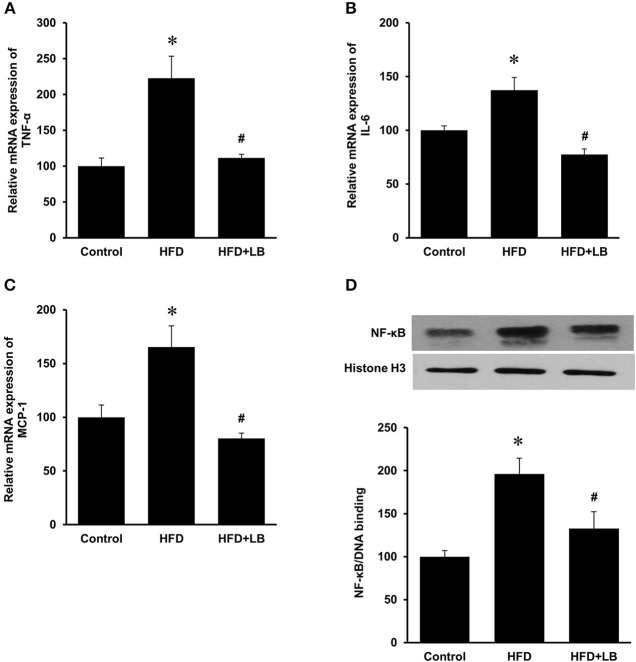
Cytokine expression and NF-κB activation in mouse kidneys. Mice were fed a control diet, high-fat diet (HFD) or HFD supplemented with lingonberry (HFD+LB) for 12 weeks. The mRNA expression of **(A)** TNF-α, **(B)** IL-6, and **(C)** MCP-1 in kidneys were measured by using qPCR analysis. **(D)** The NF-κB/DNA binding activity was determined by EMSA. Histone in the nuclear content detected by Western immunoblotting analysis served as a loading control. The results are expressed as the means ± SE (*n* = 6). *p < 0.05 when compared with the value obtained from the control group. ^#^*p* < 0.05 when compared with the value obtained from the HFD group.

**Figure 4:**
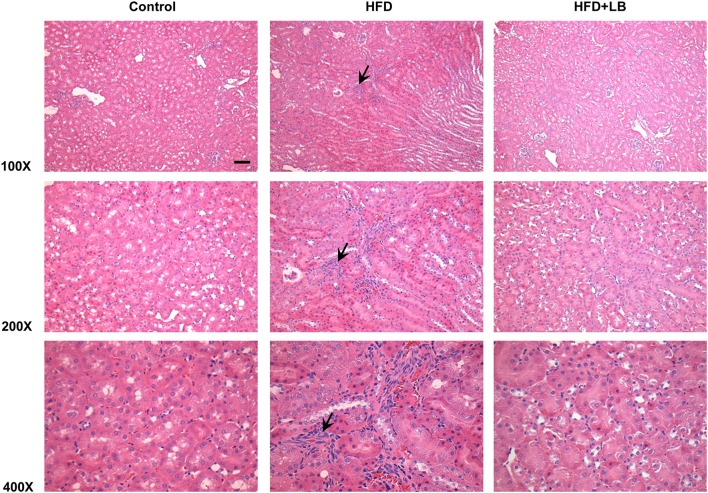
Histological staining of mouse kidneys. Mice were fed a control diet, high-fat diet (HFD) or HFD supplemented with lingonberry (HFD+LB) for 12 weeks. Kidney histology was examined by hematoxylin and eosin (H&E) staining. Arrows point to inflammatory foci (Scale bar = 100 μm).

**Figure 5:**
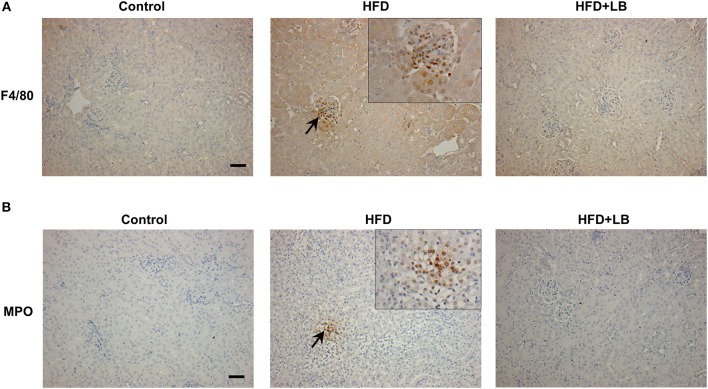
Immunohistochemical staining of mouse kidneys. Mice were fed a control diet, high-fat diet (HFD) or HFD supplemented with lingonberry (HFD+LB) for 12 weeks. Paraffin embedded kidney sections were stained with **(A)** anti-F4/80 antibody to detect macrophages or **(B)** anti-MPO antibody to detect neutrophils in the inflamed areas. Arrows point to the infiltrated macrophages and neutrophils; the images were captured at 200X. Inset: Areas containing F4/80 or MPO positive cells were enlarged 2X (Scale bar = 100 μm).

**Figure 6:**
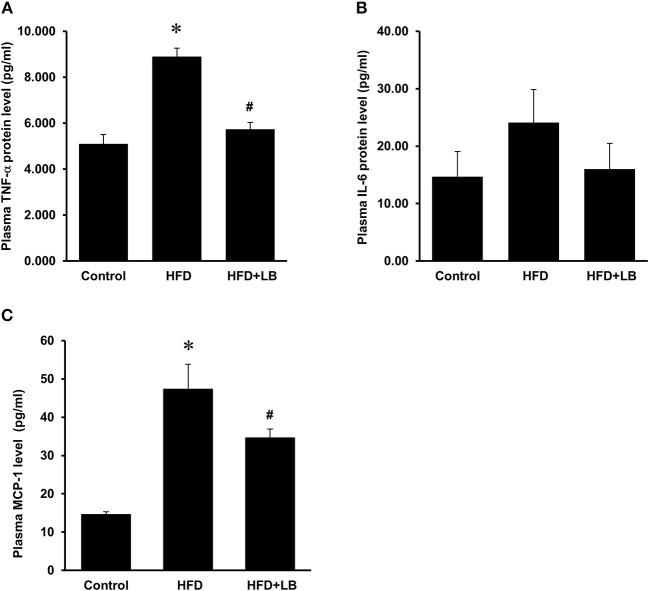
Effect of lingonberry supplementation on inflammatory cytokines in plasma of HFD-fed mice. Mice were fed a control diet, high-fat diet (HFD) or HFD supplemented with lingonberry (HFD+LB) for 12 weeks. Plasma protein levels of **(A)** TNF-α, **(B)** IL-6, and **(C)** MCP-1 were analyzed by using a multiplex assay kit. The results were expressed as the means ± SE (*n* = 4). *p < 0.05 when compared with the value obtained from the control group. ^#^*p* < 0.05 when compared with the value obtained from the HFD group.

Several proinflammatory cytokines were measured in the kidney tissue. Mice fed a HFD had a significant elevation of renal TNF-α, IL-6, and MCP-1 mRNA expression as compared to the control group (Figures 3A–C). Lingonberry supplementation attenuated the expression of these inflammatory cytokines in the kidneys (Figure 3). The transcription factor, NF-κB, is known to regulate inflammatory cytokine expression. Kidney nuclear proteins were prepared and an EMSA was carried out to determine the DNA binding activity of NF-κB. Mice fed a HFD had a significantly higher NF-κB/DNA binding activity than the control group (Figure 3D). Lingonberry supplementation effectively reduced NF-κB/DNA binding activity in the kidneys. The morphology of the kidney tissue was examined by using H&E staining. Mice fed a HFD had an increased renal deposition of inflammatory foci (characterized by dense aggregates of cells) as compared to the control group and the lingonberry supplemented group (Figure 4). To further identify the types of inflammatory cells in the kidney, immunohistochemical staining was performed (Figure 5 and Supplementary Figure 1). The majority of inflammatory cells in the kidneys of HFD-fed mice were macrophages (Figure 5A) while neutrophils (Figure 5B) were also present but to a lesser extent. These results suggested that mice fed a HFD had an increased inflammatory response in the kidney while lingonberry supplementation could alleviate HFD-induced inflammatory cytokine expression. HFD fed mice also exhibited a significant elevation of plasma TNF-α, and MCP-1 protein levels as compared to the mice fed a control diet (Figures 6A,C). There was no significant difference in plasma IL-6 levels among mice fed the different diets (Figure 6B). Lingonberry supplementation effectively reduced plasma TNF-α, and MCP-1 protein levels (Figures 6A,C).

### Induction of Inflammatory Cytokine Expression by Palmitic Acid in Kidney Proximal Tubular Cells

**Figure 7:**
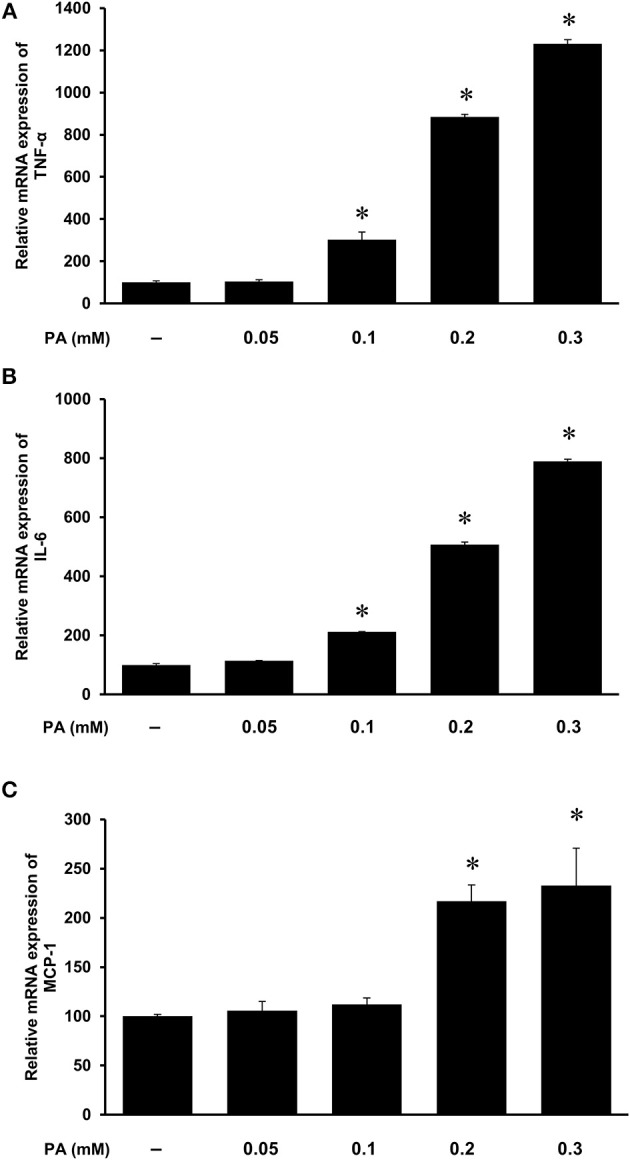
Effect of palmitic acid on cytokine expression in human proximal tubular cells. Proximal tubular cells were incubated in the absence (control) or presence of palmitic acid (PA, 0.05, 0.1, 0.2, 0.3 mM) for 4 h. The mRNA expression of **(A)** TNF-α, **(B)** IL-6, and **(C)** MCP-1 were measured by using qPCR analysis. The results are expressed as the means ± SE (*n* = 6). *p < 0.05 when compared with the value obtained from control cells.

**Figure 8:**
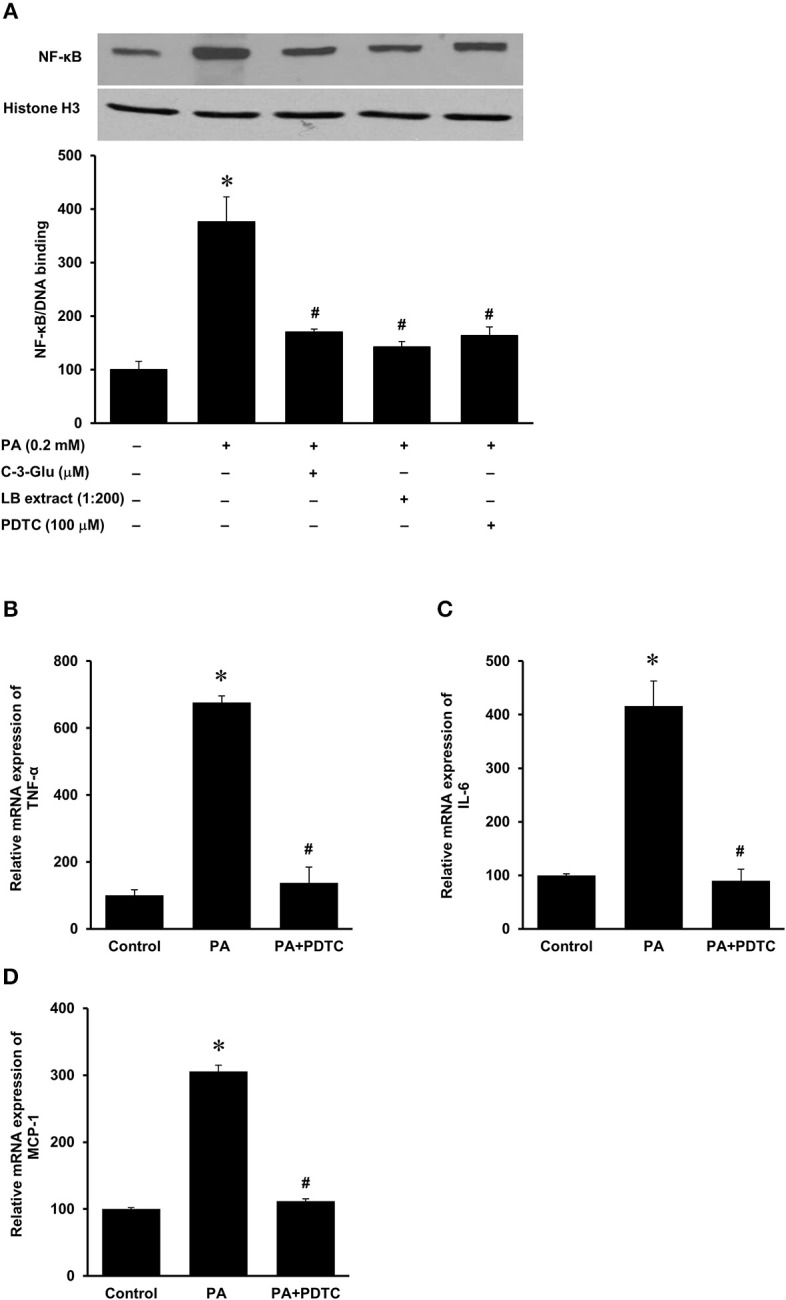
Effect of palmitic acid on NF-κB activation in human proximal tubular cells. Proximal tubular cells were preincubated with either an NF-κB inhibitor (PDTC, 100 μM) for 1 h, C-3-Glu (20 μM) for 4 h, or lingonberry (LB) extract (1:200 dilution) for 4 h, followed by incubation with palmitic acid (0.2 mM) for another 30 min. **(A)** The NF-κB/DNA binding activity was determined by EMSA. Histone in the nuclear content detected by Western immunoblotting analysis served as a loading control. In another set of experiments, cells were incubated in the absence (control) or presence of PDTC (100 μM) for 1 h, prior to incubation with palmitic acid (0.2 mM) for 4 h. The mRNA expression of **(B)** TNF-α, **(C)** IL-6, and **(D)** MCP-1 was measured by using qPCR. The results are expressed as the means ± SE (*n* = 6). *p < 0.05 when compared with the value obtained from control cells. ^#^*p* < 0.05 when compared with the value obtained from palmitic cell treated cells.

The HFD is rich in fatty acids and palmitic acid is one of the most abundant saturated fatty acids in the HFD. Palmitic acid treatment of human kidney proximal tubular cells stimulated TNF-α, IL-6, and MCP-1 mRNA expression in a dose-dependent manner (Figures 7A–C). Incubation of cells with palmitic acid also activated NF-κB (Figure 8A). To further investigate the mechanism of HFD-induced inflammatory cytokine expression, cells were incubated with an NF-κB inhibitor pyrrolidine dithiocarbamate (PDTC) (Figure 8A). Inhibition of NF-κB activation by PDTC abolished palmitic acid-induced inflammatory cytokine expression in tubular cells (Figures 8B–D).

### Effects of Lingonberry and Cyanidine-3-Glucoside on Palmitic Acid-Induced Inflammatory Cytokine Expression in Kidney Proximal Tubular Cells

**Figure 9:**
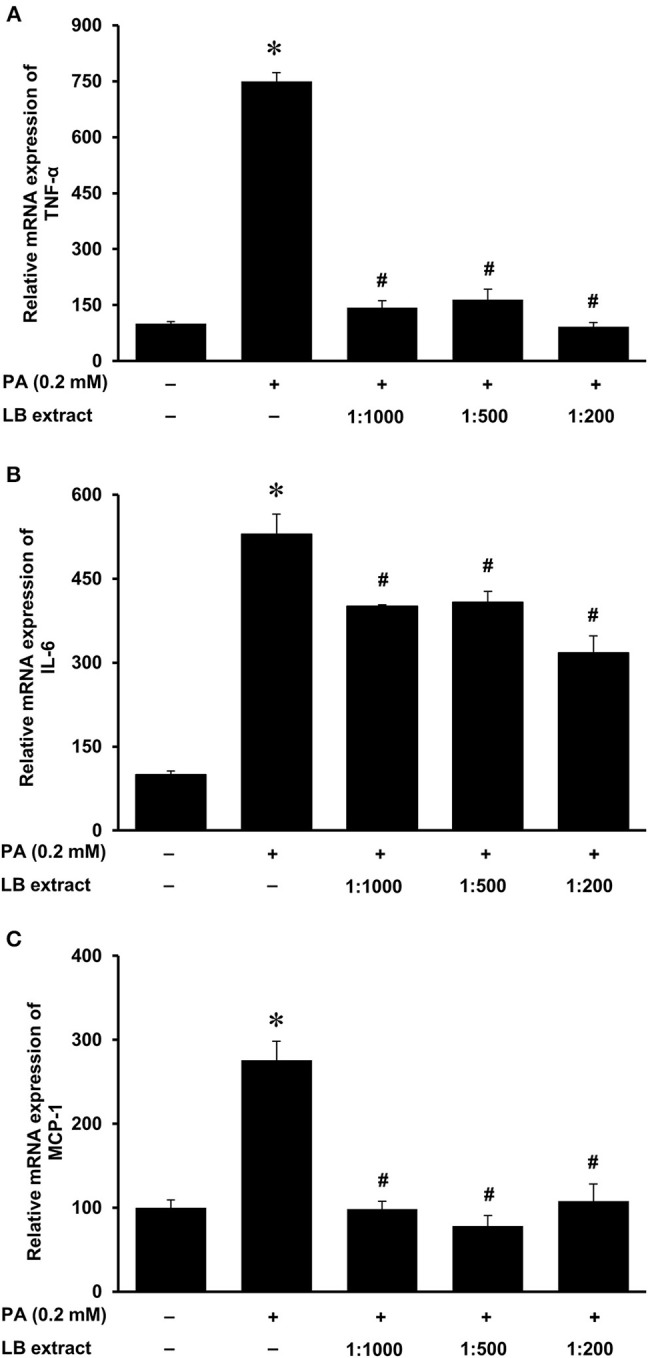
Effect of lingonberry extract and palmitic acid on cytokine expression in human proximal tubular cells. Proximal tubular cells were incubated in the absence (control) or presence of lingonberry (LB) extracts (1:1,000, 1:500, or 1:200 dilution) for 4 h followed by incubation with palmitic acid (PA, 0.2 mM) for another 4 h. The mRNA expression of **(A)** TNF-α, **(B)** IL-6, and **(C)** MCP-1 were measured by using qPCR. The results are expressed as the means ± SE (*n* = 6). *p < 0.05 when compared with the value obtained from control cells. ^#^*p* < 0.05 when compared with the value obtained palmitic acid treated cells.

**Figure 10:**
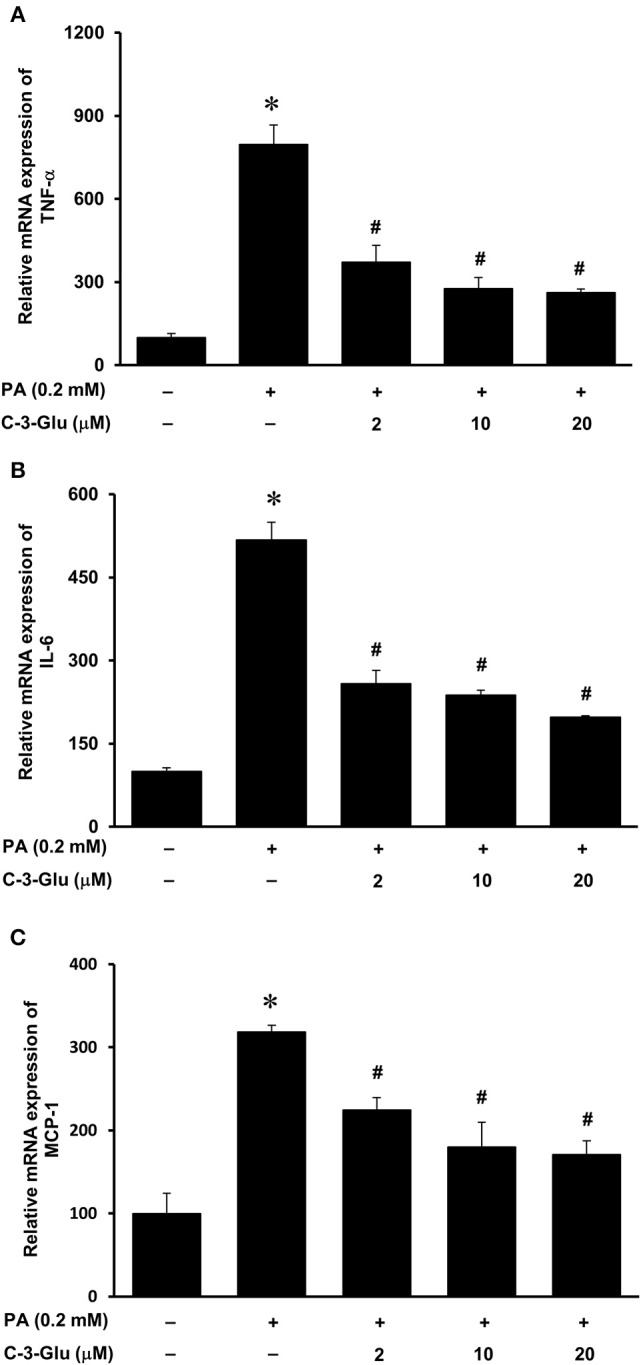
Effect of cyanidin-3-glucoside and palmitic acid on cytokine expression in human proximal tubular cells. Proximal tubular cells were incubated in the absence (control) or presence of cyanidin-3-glucoside (C-3-Glu: 2, 10 or 20 μM) for 4 h followed by incubation with palmitic acid (PA) (0.2 mM) for another 4 h. The mRNA expression of **(A)** TNF-α, **(B)** IL-6, and **(C)** MCP-1 were measured by using qPCR. The results are expressed as the means ± SE (*n* = 6). *p < 0.05 when compared with the value obtained from the control cells. ^#^*p* < 0.05 when compared with the value obtained from palmitic acid treated cells.

Next, we examined the effect of lingonberry extract or C-3-Glu on palmitic acid-induced inflammatory cytokine expression in tubular cells. C-3-Glu is one of the major lingonberry anthocyanins that have been shown to have biological activities. Incubation of cells with lingonberry extract or C-3-Glu was shown to attenuate palmitic acid-induced NF-κB activation (Figure 8A). Upon further investigation, incubation of cells with lingonberry extract significantly reduced palmitic acid-induced TNF-α, IL-6, and MCP-1 mRNA expression (Figures 9A–C, respectively). Additionally, incubation of tubular cells with C-3-Glu also effectively attenuated palmitic acid-induced inflammatory cytokine expression in a dose-dependent manner (Figures 10A–C). The palmitic acid, lingonberry extract or C-3-Glu at the doses used in the present study did not reduce cell viability.

## Discussion

Inflammatory response is one of the important mechanisms for the development of CKD (30, 31). Results obtained from the present study demonstrated that mice fed a HFD for 12 weeks developed kidney injury with increased inflammatory cytokine expression that resembled the characteristics of CKD. Lingonberry supplementation significantly reduced HFD-induced kidney injury and inflammatory response without affecting body weight gain. Our results, for the first time, showed that lingonberry extract and its anthocyanin, cyanidin-3-glucoside, could attenuate fatty acid-induced inflammatory cytokine expression in kidney tubular cells. Such an inhibitory effect was mediated through the attenuation of NF-κB activation.

Obesity and its associated metabolic abnormalities are risk factors for the development of CKD, independent of hypertension and diabetes (2). In the current study, mice fed a HFD for 12 weeks had more body weight gain as compared to the mice fed a control diet. The HFD-fed mice displayed hyperlipidemia and hyperglycemia, which resembled metabolic abnormalities seen in obese patients. HFD feeding exerted an adverse effect on kidneys as the plasma BUN levels and the expression of kidney injury biomarkers (NGAL, KIM-1, and renin) were significantly elevated. Kidney damage and inflammation can cause release of NGAL from neutrophils and kidney tubular epithelial cells (32). Overexpression of KIM-1 has also been shown to positively correlate with kidney injury (33), while inflammation and fibrosis can trigger the expression of KIM-1 at the luminal side of the proximal tubules (34). Increased tubular KIM-1 expression is an indication of ongoing tubular cell damage and dedifferentiation (35). The progression of nephron damage has also been correlated to the overexpression of renin, the initiator of renin-angiotensin axis (36). Development of renal fibrosis is a common outcome in the later stages of CKD (37). Transforming growth factor-β (TGF-β) has been identified as a key mediator of renal fibrosis (38, 39). In the current study, no significant change in TGF-β mRNA expression was observed in mice fed various diets for 12 week (see Supplementary Table 1 and Supplementary Figure 2). It is plausible that the development of renal fibrosis may become apparent in mice after a longer period of HFD feeding (40). Supplementation of lingonberry effectively prevented HFD-induced kidney injury as indicated by a decreased expression of the kidney injury biomarkers (NGAL, KIM-1, and renin) and BUN. Such a renal protective effect by lingonberry did not appear to be associated with body weight changes.

Increased expression of inflammatory cytokines contributes to the development and progression of kidney disease. In the current study, there was a significant elevation of inflammatory cytokine expression in the kidneys (TNF-α, IL-6, MCP-1) of HFD-fed mice. Histological staining and immunohistochemistry of the kidneys revealed that the predominant inflammatory cells in the HFD-fed mice were macrophages and to a lesser extent neutrophils. There was a significant elevation of inflammatory cytokine expression in proximal tubular cells after incubation with palmitic acid, the most abundant saturated fatty acid in the HFD. MCP-1 is a potent chemokine that facilitates the migration of leukocytes such as monocytes into tissues including kidneys (41–43). Upon infiltration into tissues, monocytes differentiate into macrophages that are capable of producing inflammatory cytokines such as TNF-α and IL-6 as well as releasing reactive oxygen species to aggravate tissue injury. Elevation of TNF-α and IL-6 expression are linked to increased oxidative stress, endothelial dysfunction and renal fibrosis (44). In a human study with 37 health controls and 42 CKD patients, it was reported that MCP-1 levels were significantly higher in CKD patients, especially those with glomerular disease (45). The Chronic Renal Insufficiency Cohort (CRIC) Study showed that elevated circulating levels of TNF-α was associated with progression of CKD (46). In another study (47), patients with chronic nephropathies showed an early increase in IL-6 but the levels showed no further increase with severity of CKD. In the current study, there was a significant elevation of inflammatory cytokines (TNF-α and MCP-1) in the plasma of mice fed a HFD. Lingonberry supplementation reduced these inflammatory cytokine levels in the circulation. Several lines of evidence from the current study suggested that NF-κB activation might play an important role in HFD-induced renal inflammatory response. The DNA binding activity of NF-κB was markedly increased in the kidneys isolated from HFD-fed mice as well as in palmitic acid treated tubular cells. Inhibition of NF-κB activation by its inhibitor PDTC abolished palmitic acid-induced inflammatory cytokine expression. Chronic inflammation, as exhibited by a prolonged and increased expression of inflammatory cytokines can damage kidney structure and diminish kidney function. In accordance with its protective effect on kidney function, lingonberry supplementation effectively attenuated NF-κB activation and inflammatory cytokine expression in the kidneys and plasma of mice fed a HFD. It should be noted that there was a differential effect of lingonberry extract on the expression of IL-6. This could be due to the multiple transcriptional regulation of IL-6 gene expression. Leonard et al. (48) reported that p38 and ERK MAPK pathways are important for the regulation of the production of IL-6 from the proximal tubular and glomerular mesangial regions of the nephron. Additionally, there may be potential involvement of the Jagged1-Notch signaling pathway (49). These signaling pathways may independently regulate the expression of IL-6. Aside from NF-κB, the involvement of other signaling pathways will be the focus of a separate future study. Furthermore, incubation of tubular cells with lingonberry extract or its active ingredient C-3-Glu effectively attenuated palmitic acid-induced inflammatory cytokine expression in tubular cells. C-3-Glu is one of the most extensively studied anthocyanins shown to possess various potential beneficial effects against oxidative stress and inflammation (50), insulin resistance (51), and dyslipidemia (52). Although C-3-Glu could be one of the potential bioactive compounds account for renal protective effect of lingonberry, future studies are warranted to evaluate the impact of other bioactive compounds in lingonberry. Taken together, our results suggested that attenuation of HFD-induced chronic inflammatory cytokine expression might be one of the mechanisms responsible for renal protective effect by lingonberry.

In conclusion, chronic consumption of HFD increases renal inflammatory cytokine expression and causes kidney injury. The current study, for the first time, demonstrates that dietary supplementation with lingonberry reduces HFD-induced inflammatory response and prevents kidney injury. Such a renal protective effect by lingonberry is, in part, mediated through the NF-κB signaling pathway. As current treatment option for patients with CKD is limited, lingonberry supplementation may serve as an alternative approach for the management of CKD.

## Data Availability Statement

All datasets generated for this study are included in the article/Supplementary Material.

## Ethics Statement

The animal study was reviewed and approved by University of Manitoba Protocol Management and Review Committee.

## Author Contributions

YS and KO conceived and designed research. SM and SP performed experiments. SM, SP, KO, and YS analyzed data. YS, KO, SM, and SP interpreted results of experiments. SM prepared figures. YS, KO, and SM drafted manuscript. YS, KO, SM, SP, and SD edited, revised, and approved final version of manuscript.

### Conflict of Interest

The authors declare that the research was conducted in the absence of any commercial or financial relationships that could be construed as a potential conflict of interest.
