# Supplementary material for: Inhibition of Inflammatory Cytokine Expression Prevents High-Fat Diet-Induced Kidney Injury: Role of Lingonberry Supplementation
Source: Front Med (Lausanne). 2020 Mar 27;7:80. doi: 10.3389/fmed.2020.00080 (PMC7119336; doi:10.3389/fmed.2020.00080)
Supplement: Supplementary file 1 [file Data_Sheet_1.pdf]

Supplementary Material 1

Materials and Method

Immunohistochemistry was performed on 10 μm thick frozen kidney tissue sections cut using a Leica CM1850 UV Cryostat (Wetzlar, Germany). Endogenous peroxidases were quenched in 0.3% H_2O_2 in water for 10 min. Sections were blocked in 1% BSA in PBS for 30 min followed by incubating with the primary antibodies for F4/80 (1:100 dilution, ab100790, Abcam, Cambridge, United Kingdom) or MPO (1:100 dilution, ab9535, Abcam) overnight at 4°C. The sections were washed and incubated with goat anti-rabbit IgG (1:200, Dako, Glostrup, Denmark) for 1 h at RT followed by incubation with streptavidin-horse radish peroxidase (HRP) conjugate (Zymed Laboratories, Inc., San Francisco, CA, USA). All slides were counterstained with Mayer's hematoxylin and images were captured using an Olympus BX43 Upright Light Microscope (Olympus Corp., Tokyo, Japan) equipped with a Q-color 3 digital camera and analyzed using Image-Pro plus 7.0 (Media Cybernetics, Rockville, MD, USA).

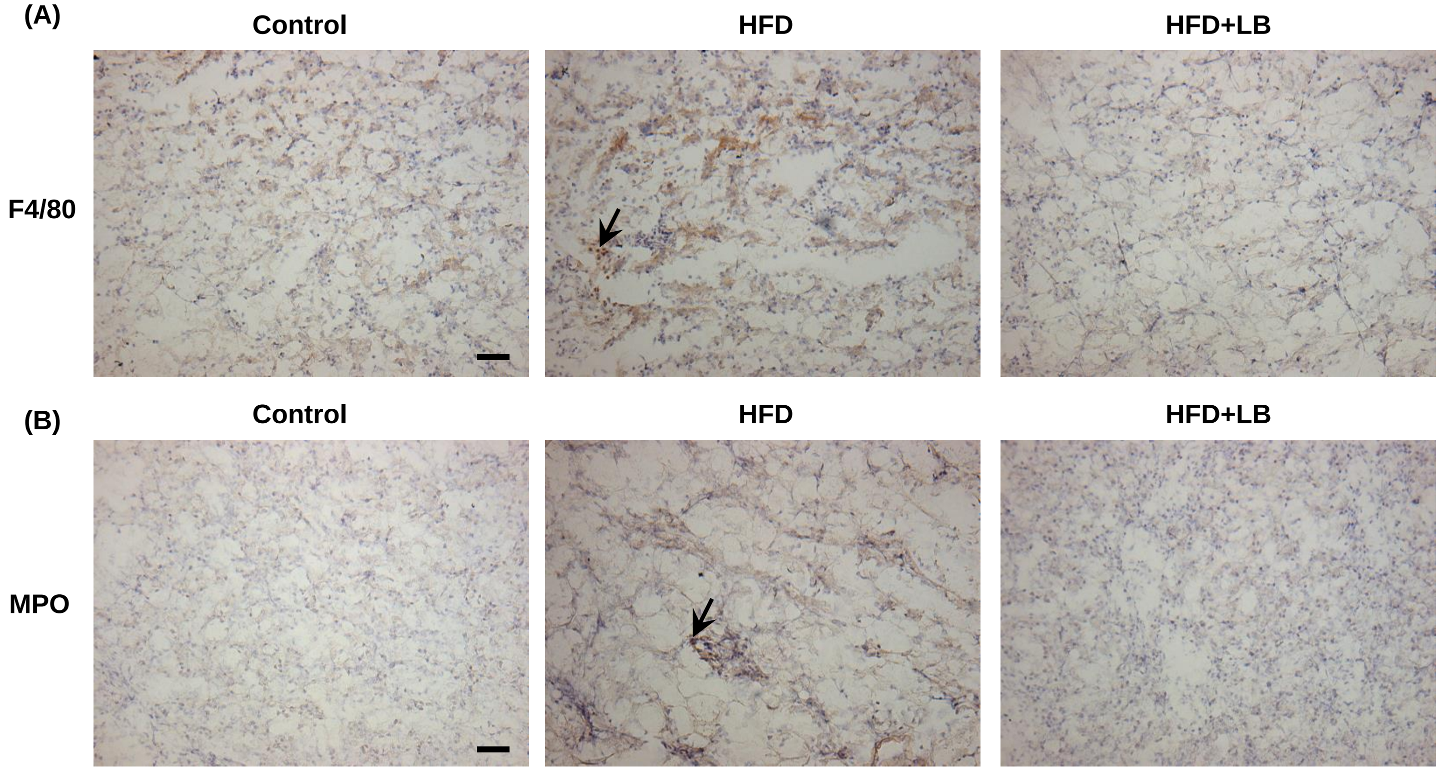

Supplementary Figure 1. Immunohistochemical staining of frozen sections of mouse kidneys.

Mice were fed a control diet, high-fat diet (HFD) or HFD supplemented with lingonberry (HFD+LB) for 12 weeks. Frozen kidney sections were stained with (A) anti-F4/80 to detect macrophages or (B) anti-MPO to detect neutrophils in the kidney tissues. Arrows point to the infiltrated macrophages and neutrophils; the images were captured at 200X. (Scale bar = 100 μm).
