# Supplementary material for: Inhibition of Inflammatory Cytokine Expression Prevents High-Fat Diet-Induced Kidney Injury: Role of Lingonberry Supplementation
Source: Front Med (Lausanne). 2020 Mar 27;7:80. doi: 10.3389/fmed.2020.00080 (PMC7119336; doi:10.3389/fmed.2020.00080)
Supplement: Supplementary file 2 [file Data_Sheet_2.pdf]

Supplementary Material 2

Materials and Method

Measurement of mRNA Expression: Relative mRNA expression of transforming growth factor beta (TGF-β) in mouse kidney tissues were measured using a StepOne Plus Real-Time qPCR (RT-qPCR) system as described in the materials and methods. The qPCR reaction mixture was subjected to initial denaturation at 95°C for 3 mins followed by 45 cycles of denaturation step 95°C, 10 s and annealing. Annealing temperature was set at 58°C for 30 s.

Supplementary Table 1. Mouse TGF-β primer sequence used for RT-qPCR

Primer	Sequence 5' - 3'	Accession Number	Product length
TGF-β	F: TACCATGCCAACTTCTGTCTGGGA	NM_011577.2	196 bp
	R: TGTTGGACAACTGCTCCACCTTG		

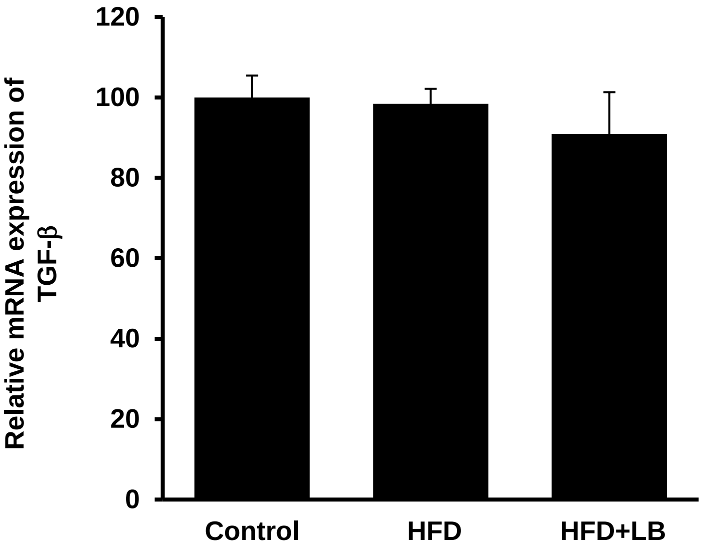

Supplementary Figure 2. Expression of transforming growth factor-β in the mouse kidneys

Mice were fed a control diet, high-fat diet (HFD) or HFD supplemented with lingonberry (HFD+LB) for 12 weeks. The mRNA expression of TGF-β in kidneys are expressed as the means ± SE (n=6). *$p < 0.05$ when compared with the value obtained from the control group. $^{\#}p < 0.05$ when compared with the value obtained from the HFD group.
